# Exploring the Awareness of Noise-Induced Hearing Loss from Headphone Use: A Cross-Sectional Study Integrating the Health Belief Model and COM-B Framework

**DOI:** 10.3390/healthcare13233059

**Published:** 2025-11-26

**Authors:** Ekramy M. Elmorsy, Mugrin Radi A. Alrwaili, Abdullah Shafi D. Alanazi, Rashed Satam B. Alshammari, Omar Mosab Alenazi, Sultan Shayish N. Alanazi, Jazzaa Hassan J. Alshammari, Manal S. Fawzy

**Affiliations:** 1 Center for Health Research, Northern Border University, Arar 73213, Saudi Arabia; ekramy.elmorsy@nbu.edu.sa; 2 Faculty of Medicine, Northern Border University, Arar 91431, Saudi Arabia; mgrenrade@gmail.com (M.R.A.A.); abdullh.shafi1@gmail.com (A.S.D.A.); stttmy@gmail.com (R.S.B.A.); smoo201079@gmail.com (O.M.A.); s6o@outlook.com (S.S.N.A.); jazza1419@gmail.com (J.H.J.A.)

**Keywords:** noise-induced hearing loss, personal listening devices, headphone use, awareness, risk factors, Saudi Arabia

## Abstract

**Highlights:**

**What are the main findings?**

- Most participants recognize the risks of noise-induced hearing loss from headphone use, but significant knowledge gaps and unsafe listening behaviors persist in the studied population.
- Behavioral model analysis (HBM and COM-B) identified insufficient knowledge, environmental factors, and inconsistent preventive actions as key barriers to effective hearing loss prevention.

**What are the implications of the main findings?**

- Public health interventions should deliver practical guidance, address contextual barriers, and prioritize at-risk groups through innovative, theory-driven campaigns.
- Device manufacturers, workplaces, and healthcare providers all play critical roles in supporting safer listening habits and promoting hearing health.

**Abstract:**

**Background/Objectives:** Noise-induced hearing loss (NIHL) is a growing public health concern, particularly with the widespread use of personal listening devices (PLDs). Limited evidence exists on NIHL awareness and risk factors in the Northern Border Region of Saudi Arabia. This study assessed awareness of NIHL associated with headphone use, identified associated risk factors and preventive attitudes, and interpreted the findings using the Health Belief Model (HBM) and the COM-B framework. **Methods:** A cross-sectional survey was conducted among 462 adults (18–60 years) using a validated online questionnaire distributed via social media. The collected data included demographics, PLD utilization, manifestations of hearing loss, knowledge, attitudes, and risk factors. Analysis included descriptive statistics and mapping results to HBM and COM-B constructs. **Results:** Among respondents, 54.3% were male, and 61.9% held a university degree. Additionally, 40.7% regularly used headphones/earphones, and 53.9% reported exposure to workplace noise. Overall, 74.7% noted at least one symptom of hearing loss, and 42.4% experienced tinnitus. Age, smoking, chronic disease, family history, workplace noise, and PLD use frequency/duration were significantly associated with hearing loss problems (all *p* < 0.05). While 78.4% recognized high-volume risk, only 58.0% believed NIHL is preventable. Social media was the primary source of information, and most participants favored device- or behavior-based interventions. Model-based analysis revealed gaps in perceived susceptibility and behavioral capability. **Conclusions:** Despite moderate general awareness, substantial knowledge gaps and unsafe listening behaviors persist. Integration of HBM and COM-B analysis highlights the need for tailored public health approaches and multifaceted NIHL prevention strategies.

## 1. Introduction

Hearing loss (HL) has emerged as a significant global public health challenge, affecting approximately 6.1% of the world’s population [1,2]. Its prevalence increases with age, making it one of the most common chronic health conditions among adults [2]. Depending on severity, HL can profoundly impair quality of life by limiting communication, causing social isolation, hindering workplace performance, and contributing to psychosocial and economic burdens [3,4,5].

Epidemiological studies highlight HL as an emerging problem among younger populations as well [6,7]. Although the etiology of HL varies with age, ranging from genetic and infectious causes in children to presbycusis and noise exposure in adults, noise-induced hearing loss (NIHL) has attracted growing attention as one of the most preventable forms of HL [8,9].

NIHL results from prolonged exposure to loud sounds, which irreversibly damage cochlear sensory hair cells [10]. Safe listening guidelines suggest that sound levels above 60 dB for more than one hour can cause temporary threshold shifts, while sustained exposure to 85 dB or higher for at least 8 h daily can lead to permanent auditory damage [11]. Traditionally, occupational noise exposure was considered the dominant risk factor; however, in recent decades, recreational noise exposure has become increasingly prevalent [12,13]. It is estimated that over 600 million individuals globally, representing more than 12% of the population, remain at risk of developing NIHL due to unsafe noise levels [12,14].

The rapid adoption of smartphones and portable listening devices (PLDs), including headphones and earphones, has amplified concerns regarding recreational noise exposure [15]. These devices enable prolonged listening at unsafe volumes, often without the user’s awareness of the associated risks. Alarmingly, improper use of headsets is linked to tinnitus, dizziness, difficulty in speech comprehension, and irreversible hearing impairment [16]. Despite these risks, public awareness about the safe use of PLD remains limited in many regions [17,18,19,20].

In Saudi Arabia, multiple studies across different regions (Southern, Makkah, Jazan, and Eastern Provinces) have identified high basic awareness of NIHL, but also revealed critical gaps in knowledge regarding harmful sound levels, exposure duration, symptoms, and safe listening habits [19,21,22,23]. Nationally, approximately 49–59% of surveyed groups demonstrate awareness of NIHL, but knowledge of safe listening limits and consistent protective behavior remains unsatisfactory [24].

International research aligns with these findings, showing that, despite basic awareness, detailed knowledge, and preventive practices, these are lacking in many populations, especially where public health campaigns are limited and PLD use is culturally normalized as part of modern communication and social enjoyment [15]. These international patterns underscore the importance of bridging knowledge gaps that are specific to each region’s population, age groups, and cultural practices [25].

Behavioral science frameworks offer critical insight into why individuals adopt or neglect preventive measures [26]. The Health Belief Model (HBM) systematically examines how perceived susceptibility, severity, benefits, and barriers influence health behaviors [27], while the COM-B model (Capability, Opportunity, Motivation–Behavior) clarifies the interaction between knowledge, environmental factors, and personal motivation in determining behavioral outcomes [28,29]. Integrating these models provides a structured framework for assessing the psychological and contextual factors that underlie the safe or risky use of personal listening devices. In the present study, the HBM and COM-B frameworks were employed not only to guide data analysis but to improve translation of findings into actionable public health strategies.

Taken together, there is a clear need for targeted public health interventions and structured educational campaigns, both globally and in Saudi Arabia, to assess and raise awareness, identify risk factors, and motivate behavioral changes to reduce the burden of NIHL from headphone use. However, deeply contextualized data from the Northern Border Region remain limited, justifying the relevance and novelty of this study in fulfilling these objectives in this population.

## 2. Materials and Methods

### 2.1. Study Design

A cross-sectional analytical study was conducted to assess adults’ awareness of noise-induced hearing loss from headphone use in the Northern Border Region of Saudi Arabia.

### 2.2. Study Population and Sampling

Eligible participants were male and female adults aged 18 to 60 years residing in the Northern Border Region, irrespective of nationality. Individuals older than 60 years were excluded to minimize confounding from age-related hearing loss. Participants were recruited between 10 September 2024 and 15 November 2024 using a convenience sampling approach. The survey link was widely disseminated across multiple social media platforms (e.g., WhatsApp groups, Twitter, Facebook) and local community groups to maximize geographic and demographic reach within the Northern Border Region. The invitation included a brief study explanation and eligibility criteria (age 18–60, residence in the region, willingness to participate). To help ensure data integrity, each respondent could submit only one entry per device, and duplicate or incomplete responses were identified and excluded during data cleaning. No financial or material incentives were provided. All participation was voluntary and anonymous, with informed consent obtained electronically before survey access.

Sample size was calculated using the Raosoft sample size calculator (Raosoft Inc., Seattle, WA, USA). Assuming a 5% margin of error, 95% confidence interval, and population response distribution of 50%, a minimum of 384 respondents was required.

### 2.3. Ethical Statement

Ethical approval for this study was obtained from the Local Bioethics Committee (HAP-09-A-043) at Northern Border University (approval no. 101/24/H) dated 6 September 2024, and the work was conducted in accordance with the principles outlined in the Declaration of Helsinki.

### 2.4. Data Collection Tool

Data were collected using a previously validated structured questionnaire adapted from relevant literature and expert consensus [30]. The survey was administered electronically via a Google Form. It consisted of 37 items (Supplementary File) divided into six major domains:

Demographic and health status characteristics data (10 items): sex, age range, nationality, residence, education level, marital status, occupation, smoking status, chronic health conditions, and family history of hearing problems.

Risk factors related to NIHL (6 items): exposure to occupational noise, preferred type of audio device, frequency and duration of listening sessions, typical volume settings, and perceived impact on surroundings.

Signs and symptoms (5 items): tinnitus occurrence, increased speech volume, repeated requests for clarification, frequent increases of TV/radio volume, and time required for acclimatization to loud environments.

Knowledge and beliefs (10 items): perceptions of how noise and environment affect hearing, recognition of early warning signs, beliefs about NIHL preventability, and awareness of sound level and exposure duration risks.

Practices and attitudes (6 items): information sources about NIHL, personal listening practices, support for device-based volume limitations, readiness for behavioral change, endorsement of warning indicators, and willingness to use sound level control programs.

Prevention awareness: views and behaviors regarding preventive measures and family protection.

### 2.5. Pilot Study and Validation

Before distributing the primary survey, a pilot study was conducted with a subset of the sample (*n* = 40) to assess reliability and clarity. The internal consistency of the questionnaire domains was assessed using Cronbach’s alpha (α = 0.89 for the total scale), and necessary revisions were made to improve comprehensibility and data integrity.

### 2.6. Operational Definitions

NIHL awareness was defined as participants’ knowledge, attitudes, and preventive behaviors related to hearing loss from prolonged or high-volume headphone use. Overall awareness levels were categorized as low (<60%), moderate (60–79%), or high (≥80%) based on mean percentage domain and total scores.

### 2.7. Data Management and Statistical Analysis

Survey responses were downloaded, coded, and entered into Microsoft Excel for initial cleaning. Statistical analysis was performed using standard statistical software (e.g., SPSS v26, IBM, Armonk, NY, USA). Descriptive statistics (frequencies, percentages, means, SDs) were used to characterize participant demographics and summarize categorical variables.

Comparative analyses used the Chi-square or Fisher’s exact tests, where appropriate, to identify associations between categorical variables.

The knowledge score was constructed by assigning one point for each correct response to nine selected objective items within the knowledge domain (questions 22–28, 30–31). Item 29 (“Do I currently have enough information concerning the danger posed by exposure to loud noise(s) on hearing ability?”) was excluded from the final score because it assessed self-perceived sufficiency of information rather than factual knowledge. Thus, the maximum possible knowledge score was 9, with an observed range of 1–9. For each included item, correct answers were defined accordingly [21,30]. Total scores were used for statistical analysis and descriptive reporting.

Independent-sample *t*-tests and a one-way ANOVA were used to compare mean knowledge scores across demographic groups, including sex, education level, and occupation. The score was treated as a continuous variable after assessing normality. Odds ratios (ORs) and corresponding 95% confidence intervals (CIs) were calculated to investigate independent risk factors of NIHL. A significance level of *p* < 0.05 was employed. Due to the exploratory nature of the study, no formal multiple-testing adjustments were implemented. The results were meticulously analyzed to minimize the likelihood of a Type I error.

### 2.8. Theoretical Frameworks and Analytical Strategy

This study prospectively adopted the HBM and COM-B framework to guide questionnaire development, data interpretation, and the design of recommendations. Quantitative results were mapped onto the constructs of each model, allowing for a multidimensional understanding of the determinants and barriers to preventive behavior. The analytic approach allowed identification of both individual cognitive factors and social or contextual influences relevant to NIHL prevention.

## 3. Results

### 3.1. Participant Characteristics

A total of 462 participants were enrolled in the study. Males accounted for 54.3% (n = 251). The majority of participants were aged 40–49 years (37.0%). Most had attained a university-level education (61.9%). About 62.8% were married, and nearly half (45.5%) were non-healthcare workers. The prevalence of smoking was 26.6%, and chronic diseases were reported in 25.1%. A family history of hearing problems was noted in 23.4% of participants (Table 1).

### 3.2. Personal Listening Device (PLDs) Utilization

Earphones and external PLDs were the most commonly preferred device types, used by 27.3% and 39.2% of participants, respectively. Additionally, 20.1% favored car PLDs, and 13.4% used headphones. More than half (53.9%) reported exposure to workplace noise. In terms of usage, 44.2% used PLDs 1–5 times per week. The most common daily usage duration was less than one hour (38.7%), while 24.7% reported 1–2 h, and smaller proportions reported higher durations. Notably, 43.7% of respondents reported complaints from others about PLD noise. The preferred sound level varied: 28.1% used 0–49% of maximum volume, and 26.8% reported never using PLDs (Table 2).

### 3.3. Self-Reported Hearing Loss Manifestations

Reported manifestations of hearing loss included ringing in the ears (42.4%), being told to speak loudly (66.3% at least sometimes), repeatedly asking others to repeat statements (71.9% at least sometimes), and a tendency to raise TV or radio volume (74.7% at least sometimes). Most participants (66.2%) required only 1 h to accommodate their hearing after PLD use (Table 3). Regarding the cumulative number of problems, 74.7% reported experiencing at least one symptom, with 45.7% experiencing two, and 6.9% all five manifestations.

### 3.4. Factors Associated with Self-Reported Hearing Loss Manifestations

Table 4 depicts that the prevalence of hearing loss-related problems was significantly associated with age (*p* < 0.0001), marital status (*p* = 0.015), occupation (*p* = 0.0041), smoking status (*p* = 0.0001), chronic disease presence (*p* = 0.0008), and family history of hearing problems (*p* = 0.0018) (Table 4). No significant association was observed with gender or educational level. Exposure to workplace noise, frequency and duration of PLD use, complaints from others about the noise of the hearing device, and higher preferred PLD sound levels were all significantly associated with a greater prevalence of hearing loss problems (all *p* < 0.001) (Table 5).

### 3.5. Multivariate Analysis

Multivariable logistic regression identified age (OR 1.21, *p* = 0.0015), smoking (OR 1.2, *p* = 0.02), chronic diseases (OR 1.52, *p* = 0.002), family history of hearing loss (OR 1.09, *p* = 0.03), workplace noise (OR 1.42, *p* = 0.003), frequency (OR 1.22, *p* = 0.007) and duration of PLD use (OR 1.63, *p* = 0.0001) as significant independent factors of hearing loss manifestations (Table 6).

### 3.6. Knowledge and Beliefs Regarding NIHL

Most participants (78.4%) acknowledged that high volume levels can affect hearing, and 66.9% recognized the influence of noisy environments. However, knowledge of specific risks was moderate: only 58% believed NIHL is preventable, and 35.3% stated they had sufficient information about the dangers of loud noise exposure. Accurate identification of minimum harmful volume and duration levels was observed in only a minority, while 60.8% did not know the minimum sound level that could negatively impact hearing (Table 7). The mean knowledge score was 6.3 ± 3.9 (range: 1–9), with no significant association found by sex, age, education, marital status, occupation, smoking status, or family history (Table 8).

### 3.7. Attitudes and Practices

Regarding information sources, 39.8% accessed knowledge from social media, followed by hospitals (24.0%) and awareness campaigns (18.0%). Most participants (87.7%) preferred reducing device volume rather than total listening time, and 68.2% agreed that manufacturers should install voice-limiting features. A substantial proportion (81.6%) reported being ready to change their behavior if presented with evidence of noise-induced risk. Most also supported installing warning indicators on audio devices and employing sound-limiting programs for household safety (Table 9).

### 3.8. Interpretation of the Study Outcomes Using Behavioral Models

The study’s outcomes were analyzed using two established behavioral frameworks: the Health Belief Model (HBM) and the COM-B model. According to the HBM, most participants (78%) recognized the general risk of loud noise exposure to hearing, but only 60.8% could identify specific harmful noise thresholds, indicating inconsistent perceived susceptibility. Although widespread reporting of hearing symptoms and an understanding of loud environments as dangerous were evident (moderate perceived severity), only 58% believed noise-induced hearing loss (NIHL) is preventable, suggesting limited perceived benefit in preventive action. A majority (87.7%) expressed a desire to reduce volume levels, and 81.6% indicated a willingness to change their behavior if provided with convincing evidence, highlighting a substantial motivation for protective practices. Key barriers included limited knowledge of safe thresholds, prevailing social norms that encouraged high-volume use, and occupational noise or device-related constraints. Social media was the predominant source of cues to action, with fewer reporting formal campaigns or peer interventions. Self-efficacy was moderate to high, with most participants confident they could act if given proper guidance and tools (Table 10).

Under the COM-B framework, psychological capability was limited by insufficient knowledge (only 60.8% aware of harmful levels); physical capability was widespread but constrained by limited access to technical controls. Physical and social opportunity barriers included high workplace noise (53.9%) and device limitations, while social influences were both positive (peer complaints) and negative (social acceptance of loud listening). Reflective motivation was strong, with a recognized risk and a willingness to change; however, habitual enjoyment of loud music reinforced risky use (Table 11).

## 4. Discussion

This cross-sectional study examined awareness, attitudes, and risk factors related to NIHL from headphone use in the Northern Border Region of Saudi Arabia, employing the HBM and COM-B framework to interpret behavioral determinants. The findings reveal substantial knowledge gaps and risky listening habits despite widespread recognition of noise exposure as a threat to hearing health.

The prevalence of hearing loss symptoms in this sample is high: 74.7% of participants reported at least one symptom, and 42.4% experienced tinnitus. These findings align with global trends indicating a rise in NIHL among young and middle-aged adults, which is associated with the increasing use of PLDs [15]. Age, smoking, chronic disease, family history, workplace noise, and both frequency and duration of PLD use were significantly associated with greater risk, consistent with previous studies highlighting these exposures as principal risk factors [22,25,31].

Knowledge assessment revealed that while 78.4% of participants recognized the harmfulness of high volume levels, a much lower proportion correctly identified specific thresholds for hazardous exposure, and only 58% believed NIHL to be preventable. This suggests that general awareness does not consistently translate into accurate risk perception or protective behavior, a pattern observed globally, especially in settings where health education on NIHL is limited [32]. The predominance of social media as an information source raises questions about the quality and reach of health literacy interventions and underlines the need for structured campaigns using reliable platforms [15,24,33,34].

Interpreting these outcomes through the HBM demonstrates inconsistent perceived susceptibility and moderate perceived severity. While symptoms were common and most participants recognized noise as a danger, many struggled to accurately assess their own behaviors or the reversibility of the harm they caused. The desire to reduce volume and the readiness to change reported by most respondents underscore strong motivation when clear benefits and cues to action are established. However, barriers such as insufficient knowledge, social normalization of high-volume listening, and environmental factors (e.g., workplace noise) hinder protective action.

The COM-B analysis further elucidates that behavioral change is constrained by limited psychological capability (knowledge deficits) and by environmental and social opportunity (prevalence of workplace noise, lack of device controls, and permissive social attitudes regarding loud PLD use). Reflective motivation for change appears robust, with most participants open to interventions if supported by tangible evidence and effective cues. In contrast, automatic motivation (habit and enjoyment) may counter efforts unless addressed in campaigns and policy.

Collectively, these results highlight the urgent need for multifaceted public health strategies. Health promotion activities should incorporate behavior change theory, deliver evidence-based, tailored messaging via trusted channels, support environmental and policy changes (such as volume-limiting features on devices), and embed NIHL prevention within broader health literacy and chronic disease frameworks. Targeted interventions for high-risk groups (smokers, those with occupational exposure) and reinforcement of preventive attitudes among youth are especially warranted.

It is worth noting that Saudi Arabia is a vast and sociodemographically diverse country, with marked variations in geography, population density, occupational risk profiles, cultural practices, and health service accessibility across its regions. While several studies have explored NIHL and hearing health awareness in regions such as Riyadh, Makkah, Jazan, and the Eastern Province, often using similar instruments for comparability, this region-specific research is crucial for several reasons: First, the burden of hearing loss, exposure risks (e.g., industrialization, prevalence of consanguinity, rural isolation), and key determinants (education, socioeconomic status, cultural attitudes) can differ markedly between regions [35]. For example, industrialized eastern regions report higher prevalence, in part due to occupational exposures. In contrast, southern and northern rural regions face unique challenges related to terrain, health literacy, and access to healthcare resources [36,37,38]. Second, regionally tailored data enables local public health and policy interventions to be adjusted effectively, ensuring that needs and service gaps are addressed in alignment with Saudi Arabia’s Vision 2030 goals [39,40].

While national-level studies provide a broad epidemiological perspective, local or regional studies, using comparable tools, offer granular insights necessary for implementation science and targeted health promotion [41]. Our study uniquely contributes data from the underrepresented Northern Border Region, supporting intra-national comparisons and province-level policy planning. The similarities in questionnaire structure across studies improve comparability. Still, our research adds specific value by documenting context-linked risk patterns, health literacy gaps, and opportunities for culturally and regionally adapted intervention in this part of Saudi Arabia [35].

### 4.1. Study Strengths and Limitations

The integration of the HBM and COM-B frameworks is a key strength, enabling a comprehensive analysis of the cognitive, social, and contextual factors that contribute to NIHL risk. The study’s limitations include reliance on self-report, a cross-sectional design that precludes causal inference, and recruitment through online platforms, which may introduce potential selection bias.

Furthermore, recruiting participants via online platforms using a convenience sampling approach from a single region may introduce significant selection bias and limit the generalizability of these findings. Individuals without internet access, lower health engagement, or from underrepresented demographic groups are likely to be excluded, leading to a sample skewed toward those more familiar with technology, with higher education, or with greater health awareness. In addition, the single-region focus restricts the applicability of our results to other geographic or cultural contexts within Saudi Arabia, where occupational, educational, or healthcare access factors may vary considerably.

It is also important to note that this study assessed hearing loss solely through self-reported symptoms rather than objective audiometric testing. Evidence indicates that self-reported hearing status shows only moderate agreement with objectively measured hearing loss, and discrepancies may vary by age, gender, and cultural context [42,43]. Self-reports can miss milder degrees of impairment or be influenced by factors such as awareness, stigma, or comorbid symptoms [44,45]. Future studies should integrate both subjective and objective (audiometric) assessments to improve diagnostic validity and comparability with broader epidemiological data.

### 4.2. Implications and Future Directions

**Figure 1:**
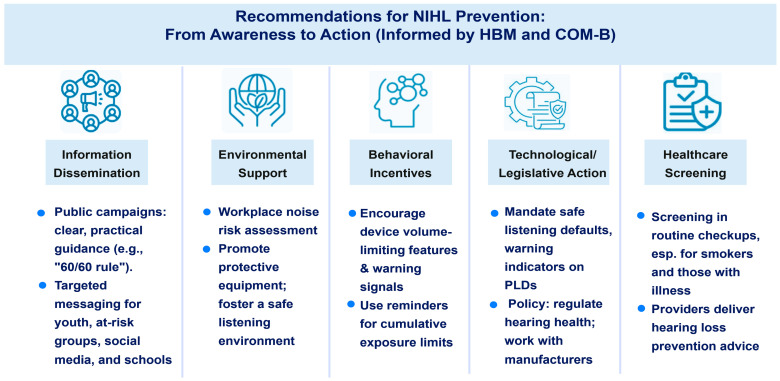
Summary of multi-level, actionable recommendations to mitigate NIHL from PLD use and occupational exposure, targeting information, environment, behaviors, technology, and healthcare, with an evidence base in HBM and COM-B frameworks.

Future research should evaluate the impact of multifaceted educational interventions and policy measures on behavior and hearing health outcomes. Collaboration among healthcare providers, policymakers, and the media is essential for achieving adequate health literacy and preventing NIHL in vulnerable populations (Figure 1).

## 5. Conclusions

This study demonstrates that although most participants recognize the general risks of NIHL associated with PLD use, significant gaps persist in specific knowledge, risk perception, and the adoption of protective behaviors. Motivation for safer practices is high if clear, practical guidance and environmental support are provided. However, barriers, including insufficient information about safe listening, technological limitations, workplace exposure, and prevailing social norms, remain substantial. These findings highlight the urgent need for multifaceted, theory-driven interventions.

## Figures and Tables

**Table 1:** Demographic and history-related data for the study participants.

Parmeter	Number	%
Sex		
Males	251	54.3
Females	211	45.7
Age		
18–25	108	23.4
26–39	114	24.7
40–49	171	37.0
50–60	69	14.9
Educational level		
Pre-university	176	38.1
University	286	61.9
Marital status		
Widow	13	2.8
Single	142	30.7
Married	290	62.8
Divorced	17	3.7
Job		
Student	70	15.2
Non-medical	210	45.5
Healthcare worker	58	12.6
Not working	124	26.8
Smoking		
No	339	73.4
Yes	123	26.6
Chronic diseases		
HTN	54	11.7
Cardiac	11	2.4
DM	51	11.0
No	346	74.9
Family history of hearing problems		
No	354	76.6
Yes	108	23.4

Data are presented as numbers and percentages (%). HTN: hypertension, DM: diabetes mellitus.

**Table 2:** Participants’ personal listening device (PLD) utilization-related data.

Questions	Answers Numbers	%
Preferred device type		
Headphone	62	13.4
Car PLDs	93	20.1
External PLDs	181	39.2
Earphone	126	27.3
Noise in the workplace		
No	213	46.1
Yes	249	53.9
Using times per week		
Never	124	26.8
1–5	204	44.2
6–9	77	16.7
10	57	12.3
Daily using duration (hours)		
<1	179	38.7
1–2	114	24.7
2–5	20	4.3
>5	25	5.4
Never	124	26.8
Others are complaining of my PLD noise		
Never	174	37.7
Sometimes	202	43.7
Often	60	13.0
Always	26	5.6
Preferred PLDs sound level		
Never used PLDs	124	26.8
0–49	130	28.1
50–59	100	21.6
60–69	98	21.2
70–79	51	11.0
80–89	18	3.9
90–100	65	14.1

Data are presented as numbers and percentages (%). PLDs: personal listening devices.

**Table 3:** Self-reported hearing loss manifestations among the participants.

Questions	Answers Numbers	%
Feeling ringing in the ear		
No	266	57.6
Yes	196	42.4
Others say that you are speaking loudly		
Never	147	31.8
sometimes	98	21.2
Often	175	37.9
Always	42	9.1
Asking others to repeat what they have said		
Never	130	28.1
sometimes	218	47.2
Often	87	18.8
Always	27	5.8
Prefer to raise the TV and radio sound levels		
Never	117	25.3
sometimes	222	48.1
Often	92	19.9
Always	31	6.7
Time taken to accommodate the surrounding sound after using PLDs		
1	306	66.2
5	110	23.8
10	24	5.2
15	22	4.8

Data are presented as numbers and percentages (%). TV: television, PLDs: personal listening devices.

**Table 4:** Effect of demographic factors on self-reported hearing loss among participants.

Parameters	Reported Hearing Loss Problems		No Reported Hearing Loss Problems		Total		*p*-Value
	*n*	%	*n*	%	*n*	%	
Sex							
Females	165	78.2	46	21.8	211	100	0.132 ^a^
Males	180	71.7	71	28.3	251	100	
Age							
18–25	66	61.1	42	38.9	108	100	<0.0001 ***
26–39	70	61.4	44	38.6	114	100	
40–49	151	88.3	20	11.7	171	100	
50–60	58	84.1	11	15.9	69	100	
Educational level							
Pre-university	135	76.7	41	23.3	176	100	0.43 ^a^
University	210	73.4	76	26.6	286	100	
Marital status							
Widow	8	61.5	5	38.5	13	100	0.015 *
Single	117	82.4	25	17.6	142	100	
Married	211	72.8	79	27.2	290	100	
Divorced	9	52.9	8	47.1	17	100	
Job							
Student	43	61.4	27	38.6	70	100	0.0041 **
Non-healthcare jobs	171	81.4	39	18.6	210	100	
Healthcare worker	39	67.2	19	32.8	58	100	
Not working	92	74.2	32	25.8	124	100	
Smoking							
No	269	79.4	70	20.6	339	100	0.0001 *** ^a^
Yes	76	61.8	47	38.2	123	100	
Chronic diseases							
HTN	48	88.9	6	11.1	54	100	0.0008 ***
Cardiac	9	81.8	2	18.2	11	100	
DM	46	90.2	5	9.8	51	100	
No	242	69.9	104	30.1	346	100	
FH hearing problems							
No	252	71.2	102	28.8	354	100	0.0018 **
Yes	93	86.1	15	13.9	108	100	

Data are presented as numbers and percentages (%). ^a^ Fisher’s exact test, * *p*-value < 0.05, ** *p*-value < 0.01, *** *p*-value < 0.001. HTN: hypertension, DM: diabetes mellitus, FH: family history.

**Table 5:** Effect of the other risk factors studied on hearing loss problems among the participants.

Parameters	Hearing Loss Problem		No Hearing Loss Problem		Total		*p*-Value
	*n*		%	*n*	*n*	%	
Noise in the workplace							
No	143	67.1	70	32.9	213	100	0.0006 *** ^a^
Yes	202	81.1	47	18.9	249	100	
Using times per week							
Never	68	54.8	56	45.2	124	100	<0.0001 ***
1–5	165	80.9	39	19.1	204	100	
6–9	63	81.8	14	18.2	77	100	
10	49	86.0	8	14.0	57	100	
Daily duration (hours)							
<1	140	78.2	39	21.8	179	100	<0.0001 ***
1–2	97	85.1	17	14.9	114	100	
2–5	15	75.0	5	25.0	20	100	
>5	21	84.0	4	16.0	25	100	
Never	72	58.1	52	41.9	124	100	
Others are complaining of my PLD noise							
Never	107	61.5	67	38.5	174	100	<0.0001 ***
Sometimes	164	81.2	38	18.8	202	100	
Often	52	86.7	8	13.3	60	100	
Always	22	84.6	4	15.4	26	100	
Preferred PLD sound level							
Never used PLDs	30	24.2	94	75.8	124	100	<0.0001 ***
0–49	83	63.8	47	36.2	130	100	
50–59	56	56.0	44	44.0	100	100	
60–69	72	73.5	26	26.5	98	100	
70–79	43	84.3	8	15.7	51	100	
80–89	11	61.1	7	38.9	18	100	
90–100	50	76.9	15	23.1	65	100	

Data are presented as numbers and percentages (%). ^a^ Fisher’s exact test, *** *p*-value < 0.001. PLDs: personal listening devices.

**Table 6:** Multi-logistic regression analysis data model analysis of the effect of the different parameters on the self-reported hearing problems.

Risk Factor	*p*-Value	Odds Ratios	95% Confidence Interval
Sex	0.98	0.87	0.54–1.3
Age	0.002 **	1.21	1.07–1.36
Educational level	0.76	0.57	0.32–1.03
Marital status	0.86	0.37	0.12–1.14
Job	0.37	0.58	0.33–1.06
Smoking	0.02 *	1.20	1.03–1.56
Chronic diseases	0.002 **	1.52	1.20–1.89
Family history of hearing loss	0.03 *	1.09	1.01–1.31
Noise work area	0.003 **	1.42	1.10–1.78
Times of use of PLDs	0.007 **	1.22	1.05–2.20
Duration of use of PLDs	0.0001 ***	1.63	1.30–2.10
PLDs sound annoying to the surrounding persons	0.12	0.92	0.69–1.3
Preferred PLD sound level	0.08	1.05	0.86–1.43

* *p*-value < 0.05, ** *p*-value < 0.01, *** *p*-value < 0.001. PLD: portable listening devices.

**Table 7:** Distribution of beliefs and knowledge about noise-induced hearing loss among the participants.

Do high volume levels affect hearing?		
Yes	362	78.4
No	57	12.3
I don’t know	43	9.3
Does living or working in a noisy environment affect hearing?		
Yes	309	66.9
No	91	19.7
I don’t know	62	13.4
Hearing impairment could get worse by listening to loud sound		
Yes	303	65.6
No	79	17.1
I don’t know	80	17.3
Does the hearing of low/muffled voices during daily conversation indicate the early signs of hearing impairment?		
Yes	203	43.9
No	120	26.0
I don’t know	139	30.1
Is the sensation of ringing in the ear a sign of a hearing impairment?		
Yes	166	35.9
No	207	44.8
I don’t know	89	19.3
Does frequently increasing TV or radio volume indicate a sign of hearing impairment?		
Yes	234	50.6
No	109	23.6
I don’t know	119	25.8
Are noise-induced hearing problems preventable?		
Yes	268	58.0
No	75	16.2
I don’t know	119	25.8
Do I currently have enough information concerning the danger posed by exposure to loud noise(s) on hearing ability?		
Yes	163	35.3
No	159	34.4
I don’t know	140	30.3
The minimum volume level that could negatively affect hearing is		
20–40	67	14.5
41–60	45	9.7
61–80	43	9.3
81–90	21	4.5
91–100	5	1.1
I do not know	281	60.8

Data are presented as numbers and percentages (%).

**Table 8:** Means of knowledge scores and the studied parameters.

Parmeter	Knowledge Scores		*p*-Value
	Means	SD	
Sex			
Females	6.5	2.3	0.87 ^a^
Males	6.3	3.1	
Age			
18–25	6.9	3.2	0.58 ^b^
26–39	6.3	2.6	
40–49	6.5	2.9	
50–60	5.2	1.9	
Educational level			
Pre-university	5.2	3.4	0.29 ^a^
University	6.9	3.5	
Marital status			
Widow	5.7	3.5	0.94 ^b^
Single	6.2	3.7	
Married	6.4	3.2	
Divorced	6.7	4	
Job			
Student	6.6	3.8	0.90 ^b^
Non-medical	6.4	3.9	
Healthcare worker	7.2	3.5	
Not working	5.9	4	
Smoking			
No	6.4	3.7	0.65 ^a^
Yes	6.2	4.1	
Chronic diseases			
HTN	5.9	3.2	0.73 ^b^
Cardiac	5.4	2	
DM	6.3	3.6	
No	6.5	2.9	
Family history of hearing problems			
No	6.4	3.4	0.94 ^a^
Yes	6.6	3.6	

Data are presented as mean ± standard deviation (SD). ^a^ *t*-test analysis, ^b^ one-way ANOVA analysis. HTN: hypertension, DM: diabetes mellitus.

**Table 9:** Participants’ attitude towards noise-related hearing loss.

Questions/Answers	Numbers	%
Typically accessed source of information about NIHL		
Social media	184	39.8
Media	51	11.0
Hospitals	111	24.0
Awareness campaign	83	18.0
Schools and relatives	33	7.1
Do I prefer to decrease the volume of my device over the total time of listening?		
Yes	405	87.7
No	57	12.3
I recommend that the factory install a voice-limiting feature on my PLD.		
Yes	315	68.2
no	147	31.8
I’m ready to change my behavior if I hear/see evidence that suggests that loud noise/sound levels affect hearing.		
Never	88	19.0
Sometimes	188	40.7
Often	80	17.3
Always	106	22.9
I recommend putting warning indicators on audio devices to limit volume levels.		
Yes	373	80.7
No	89	19.3
I prefer using a program to limit sound levels for me and my family.		
Never	109	23.6
Sometimes	206	44.6
Often	82	17.7
Always	65	14.1

Data are presented as numbers and percentages (%).

**Table 10:** Summary of HBM constructs and outcomes.

HBM Construct	Definition	Application Example (Noise-Induced Hearing Loss)	Expected Outcome
Perceived Susceptibility	Belief about the likelihood of being affected by a condition	Workers believe they are at risk of NIHL if exposed to loud noise	Increases motivation for preventive actions
Perceived Severity	Belief about the seriousness of the condition and its consequences	Understanding that NIHL can cause permanent hearing damage and social limitations	Reinforces urgency to adopt protective behaviors
Perceived Benefits	Belief in the effectiveness of taking preventive action	Using ear protection reduces the risk of NIHL	Encourages adherence to protective measures
Perceived Barriers	Belief about obstacles to performing preventive actions	Discomfort of earplugs, lack of workplace enforcement	May reduce compliance unless addressed
Cues to Action	External or internal triggers that stimulate action	Health campaigns, device warnings, peer encouragement	Initiates behavior change
Self-Efficacy	Confidence in the ability to take preventive action	The worker feels capable of consistently using hearing protection	Sustains protective behaviors over time
Outcome	Result of applying HBM constructs	Adoption of protective hearing behaviors → Reduction in noise-induced hearing loss	Improved long-term hearing health

**Table 11:** Summary of COM-B framework constructs and outcomes.

COM-B Construct	Definition	Application Example (Noise-Induced Hearing Loss/PLD Use)	Expected Outcome
**Capability** (Psychological & Physical)	Knowledge, skills, and the ability to perform the behavior	Many participants lack knowledge of safe listening thresholds (60.8% do not know the harmful level); they are physically able to adjust volume or use earplugs	Improving knowledge and providing tools increases safe listening behaviors
**Opportunity** (Social & Physical)	External factors that enable or hinder behavior	Workplace noise exposure (53.9%), device settings, and social norms about loud music	Reducing environmental barriers (e.g., workplace controls, device volume limits) enables safer habits.
**Motivation** (Reflective & Automatic)	Internal processes that energize or direct behavior	High readiness to change (81.6%), but the enjoyment/habit of loud music sustains risky use	Targeted cues and incentives can shift motivation toward protective actions
**Behavior**	Resulting action from the interaction of C, O, and M	Actual PLD listening patterns (frequency, duration, volume) and protective practices	Safer listening behaviors → reduced NIHL risk

These model-based interpretations offer a structured understanding of the behavioral drivers and barriers to NIHL prevention in this population.

## Data Availability

The original contributions presented in this study are included in the article and Supplementary Material. Further inquiries can be directed to the corresponding author.

## Supplementary Materials

The following supporting information can be downloaded at: https://www.mdpi.com/article/10.3390/healthcare13233059/s1, The study questionnaire in English.

## Abbreviations

The following abbreviations are used in this manuscript:

CI	Confidence interval
COM-B	Capability, Opportunity, Motivation–Behavior
DM	Diabetes mellitus
HBM	Health Belief Model
HTN	Hypertension
NIHL	Noise-induced hearing loss
OR	Odds ratio
PLD	Personal listening device
SPSS	Statistical Package for the Social Sciences
