# Supplementary material for: Exploring the Awareness of Noise-Induced Hearing Loss from Headphone Use: A Cross-Sectional Study Integrating the Health Belief Model and COM-B Framework
Source: Healthcare (Basel). 2025 Nov 26;13(23):3059. doi: 10.3390/healthcare13233059 (PMC12692397; doi:10.3390/healthcare13233059)
Supplement: Supplementary file 1 [file healthcare-13-03059-s001.zip › healthcare-3940759-supplementary.pdf]

Awareness of Noise-Induced Hearing Loss from Headphone Use: A Cross-Sectional Study

You are invited to participate in this study to assess the awareness of noise-induced hearing loss associated with headphone use among the general population.

- **Your participation** will include completing a brief questionnaire (about 5-7 minutes).

- **Participation is voluntary**, and you can withdraw from this study anytime.

- **Confidentiality:** All information collected during the study will be kept strictly confidential. Your name and identifying information will not be associated with your responses.

- **The data** will be used for research purposes only and will not be shared with anyone outside the research team.

- **Risks and Benefits:** There are no known risks associated with participating in this study. You may benefit from increasing your awareness of noise-induced hearing loss associated with headphone use.

Agree to participate in this study

- o Yes
- o No

I- The demographics of the study participants-related items

1. Gender:

- [ ] Male
- [ ] Female

2. Age:

- [ ] 18-25 years old
- [ ] 26-39 years old
- [ ] 40-49 years old
- [ ] 50-60 years old

3. Nationality:

- [ ] Saudi
- [ ] Non-Saudi

4. Place of Residence:

- [ ] Northern Region
- [ ] Other Region

5. Educational Level:

- [ ] Uneducated
- [ ] Primary
- [ ] Intermediate
- [ ] Secondary
- [ ] Diploma
- [ ] Bachelor's
- [ ] Postgraduate

6. Marital Status:

- [ ] Single
- [ ] Married

- [ ] Divorced

- [ ] Widowed

7. Occupation

- [ ] Student

- [ ] Health care worker

- [ ] non-health care worker

- [ ] Not working

8. Smoking

- [ ] Yes

- [ ] No

9. Chronic health problems

- [ ] None

- [ ] Diabetes mellitus

- [ ] Hypertension

- [ ] Cardiac

10. Family history of hearing problems

- [ ] Yes

- [ ] No

II. Risk factors related to noise induced hearing loss

11. Expose to noise at work setting or environment

- [ ] Yes

- [ ] No

12. Preferred type of audio device

[ ] Earphones
[ ] External PADs
[ ] Car PADs
[ ] Headphones

13. Number of hearing sessions per week

[ ] Never
[ ] 1–5
[ ] 6–9
[ ] 10+

14. Duration of the listening session/per day (hour)

[ ] <1
[ ] 1–2
[ ] 3–5
[ ] > 5

15. How often are the people surrounding me affected by the noise from my PAD?

[ ] Never
[ ] Sometimes
[ ] Usually
[ ] Always

16. Typical level of volume used (%)

[ ] 0–49
[ ] 50–59
[ ] 60–69
[ ] 70–79
[ ] 80–89
[ ] 90–100

III. Signs and symptoms related to noise induced hearing loss

17. Do you feel ringing in the ears?

[ ] Never
[ ] Sometimes
[ ] Usually
[ ] Always

18. People said I talk loud

[ ] Never
[ ] Sometimes
[ ] Usually
[ ] Always

19. I tend to ask "What?" repeatedly in a conversation

[ ] Never
[ ] Sometimes
[ ] Usually
[ ] Always

20. Increasing the volume of the TV or radio is something I do

[ ] Never
[ ] Sometimes
[ ] Usually
[ ] Always

21. The time I need to adapt to the surrounding environmental sound when exposed to loudness (hours)

[ ] 1 h
[ ] 5 hs
[ ] 10 hs
[ ] 15 hs

<u>IV. Beliefs and knowledge about noise-induced hearing loss</u>

22. Do high volume levels affect hearing?

- [ ] Yes

- [ ] No

- [ ] I Don't know

23. Does living or working in a noisy environment affect hearing?

- [ ] Yes

- [ ] No

- [ ] I Don't know

24. Hearing impairment could get worse by listening to loud sound

- [ ] Yes

- [ ] No

- [ ] I Don't know

25. Does the hearing of low/muffled voices during daily conversation indicate the early signs of hearing impairment?

- [ ] Yes

- [ ] No

- [ ] I Don't know

26. Is the sensation of ringing in the ear a sign of a hearing impairment?

- [ ] Yes

- [ ] No

- [ ] I Don't know

27. Does the frequent increase in TV or radio volume indicate a sign of hearing impairment?

- [ ] Yes

- [ ] No

- [ ] I Don't know

28. Are noise-induced hearing problems preventable?

- [ ] Yes

- [ ] No

- [ ] I Don't know

29. Do I currently have enough information concerning the danger posed by exposure to loud noise(s) on hearing ability?

- [ ] Yes

- [ ] No

- [ ] I Don't know

30. The minimum duration of listening to a loud noise source that could negatively affect one's hearing is

- [ ] Yes

- [ ] No

- [ ] I Don't know

31. The minimum volume level that could negatively affect hearing is

[ ] 20–40
[ ] 41–60
[ ] 61–80
[ ] 81–90
[ ] 91–100
[ ] Don't know

V. Practices and attitudes toward noise-induced hearing loss

32. A typically accessed source of information about NIHL

[ ] Social media
[ ] Hospitals
[ ] Educational campaigns
[ ] Schools and environment
[ ] Mass media
[ ] Commercial centers

33. Do I prefer to decrease the volume of my device over the total time of listening?

- [ ] Yes

- [ ] No

34. I recommend that the factory should install a voice-limiting feature on my PAD

- [ ] Yes

- [ ] No

35. I'm ready to change my behavior if I hear/see evidence that suggests that loud noise/sound levels affect hearing

[ ] Never
[ ] Sometimes
[ ] Usually
[ ] Always

36. I recommend putting warning indicators on audio devices to limit volume levels

- [ ] Yes

- [ ] No

37. I prefer using a program to limit sound levels for me and my family

[ ] Never
[ ] Sometimes
[ ] Usually
[ ] Always

References: Abdulaziz S. AlQahtani, Ahmed N. Alshammari, Eyad M. Khalifah, Akram A. Alnabri, Hadi A. Aldarwish, Khaled F. Alshammari, Hamad F. Alshammari, Abdulkarim M. Almudayni. Awareness about the relation of noise-induced hearing loss and use of headphones at Hail region, Annals of Medicine and Surgery; 73, 2022.
